# Photocatalytic Performance Improvement by Doping Ag on ZnO/MWCNTs Nanocomposite Prepared with Pulsed Laser Ablation Method Based Photocatalysts Degrading Rhodamine B Organic Pollutant Dye

**DOI:** 10.3390/membranes12090877

**Published:** 2022-09-11

**Authors:** Tahani A. Alrebdi, Reham A. Rezk, Shoug M. Alghamdi, Hoda A. Ahmed, Fatemah H. Alkallas, Rami Adel Pashameah, Ayman M. Mostafa, Eman A. Mwafy

**Affiliations:** 1 Department of Physics, College of Science, Princess Nourah bint Abdulrahman University, Riyadh 11671, Saudi Arabia; 2 Higher Technological Institute, 10th of Ramadan City, 6th of October Branch, 3rd Zone, 7th Section, 6th of October City, 10th of Ramadan 44629, Egypt; 3 Department of Physics, Faculty of Science, Taibah University, Yanbu 46423, Saudi Arabia; 4 Department of Chemistry, Faculty of Science, Cairo University, Cairo 12613, Egypt; 5 Chemistry Department, College of Sciences, Taibah University, Yanbu 46423, Saudi Arabia; 6 Department of Chemistry, Faculty of Applied Science, Umm Al-Qura University, Makkah 24230, Saudi Arabia; 7 Spectroscopy Department, Physics Division, National Research Centre, 33 El Bohouth st. (Former El Tahrir st.), Dokki, Giza 12622, Egypt; 8 Laser Technology Unit, Center of Excellent for Advanced Science, National Research Centre, 33 El Bohouth st. (Former El Tahrir st.), Dokki, Giza 12622, Egypt; 9 Physical Chemistry Department, Advanced Materials Technology and Mineral Resources Research Institute, National Research Centre, Dokki, Giza 12622, Egypt

**Keywords:** nanocomposite, NPs, water treatment, dye, laser ablation, Rhodamine B

## Abstract

ZnO/MWCNTs nanocomposite has significant potential in photocatalytic and environmental treatment. Unfortunately, its photocatalytic efficacy is not high enough due to its poor light absorbance and quick recombination of photo-generated carriers, which might be improved by incorporation with noble metal nanoparticles. Herein, Ag-doped ZnO/MWCNTs nanocomposite was prepared using a pulsed laser ablation approach in the liquid media and examined as a degradable catalyst for Rhodamine B. (RhB). Different techniques were used to confirm the formation of the nanostructured materials (ZnO and Ag) and the complete interaction between them and MWCNTs. X-ray diffraction pattern revealed the hexagonal wurtzite crystal structure of ZnO and Ag. Additionally, UV-visible absorption spectrum was used to study the change throughout the shift in the transition energies, which affected the photocatalytic degradation. Furthermore, the morphological investigation by a scanning electron microscope showed the successful embedding and decoration of ZnO and Ag on the outer surface of CNTs. Moreover, the oxidation state of the formed final nanocomposite was investigated via an X-ray photoelectron spectrometer. After that, the photocatalytic degradations of RhB were tested using the prepared catalysts. The results showed that utilizing Ag significantly impacted the photo degradation of RhB by lowering the charge carrier recombination, leading to 95% photocatalytic degradation after 12 min. The enhanced photocatalytic performance of the produced nanocomposite was attributed to the role of the Ag dopant in generating more active oxygen species. Moreover, the impacts of the catalyst amount, pH level, and contact time were discussed.

## 1. Introduction

Due to the industry’s regular activities, dye pollution is one of the world’s most serious environmental problems. Paper printing, textile dyeing, cosmetics, and pharmaceuticals are just few industries that use synthetic colors. About 20% of the entire world’s dye manufacturing is wasted during the dyeing process. One of these dyes, Rhodamine B (RhB), a common dye used in industrial applications, can irritate the skin and eyes, as well as the gastrointestinal and respiratory tracts. Dye pollution is the easiest to discover because it can be noticed by the naked eye at very low levels. Furthermore, color inhibits the passage of light into the water, which has an adverse effect on the photosynthetic process that supports the living organisms’ environment under the water [1,2,3,4,5].

To overcome these hazard compounds, physical adsorption, chemical oxidation, and reverse osmosis membrane treatment processes are some of the physical and chemical techniques that can successfully remove dye pollutants from water. However, the cost of these techniques is very high, and there are some practical issues, such as the need to use many chemicals and incompletely degraded products produced during the oxidation process; therefore, these methods cannot be used to treat actual dye wastewater on a wide scale due to numerous practical issues, such as the disposal of a lot of adsorbent residue, expensive membrane treatment equipment, and operation costs. The catalytic reduction process shows a great achievement in the field of water treatment with the assistance of nanostructured materials for a fast reduction or oxidation reactions. This method is based on converting the hazard’s organic form to a nontoxic form that is capable of being safely degradable in the water without causing any side effects to the environment. When nanostructured materials are used as catalytic degradable materials, two ways could be used to achieve the reduction. One of these ways is the photocatalytic degradation method, which is based on generating powerful hydroxyl (OH) and superoxide (^•^O_2_) radicals. These radicals can non-selectively oxidize all types of organic molecules present in wastewater. The other method is the oxidation reduction catalytic degradation method. The photocatalytic technique has emerged as one of the possible processes for treating dye wastewater due to the benefits of low cost and ease of use. Therefore, it will be crucial to create new catalysts able to absorb sunshine or other forms of visible light that are inexpensive, simple to prepare, and available in a variety of materials in order to address the energy crisis and advance pollution prevention [6,7,8,9,10,11,12].

Recently, there have been significant advances in the field of nanotechnology, many of which have speeded it up. According to their types, procedures, morphology, size, crystal structures, and other characteristics, researchers are generating a diversity of nanomaterials with improved properties. Metals, their oxides (MOs), nano-graphenes, carbon nanotubes, and nano-composites are the most important nanomaterials that may be manufactured in a variety of ways with extreme precision. These nanomaterials have incredibly controlled size, larger surface area, easy fabrication methods, and unique features and uses, and they could be prepared chemically, physically, and through biological approaches [13,14,15,16,17]. For the degradation of organic dyes, several metal oxide semiconductors have been utilized, including titanium dioxide, ferric oxide, cadmium oxide, magnesium oxide, zinc oxide (ZnO), etc. Since ZnO has a wide band gap (3.37 eV) and a high excitonic binding energy, it has been regarded as an effective and non-toxic photocatalyst. Due to its safe performance, nanostructured ZnO has been widely employed in bio-imaging, drug delivery, antibacterial, biosensors, chemosensors, anticancer, solar cell applications, and photocatalysts. ZnO is a potential material for photocatalytic degradation of water contaminants in the field of water treatment, having exceptional activity, low cost, and environmentally acceptable features. After being exposed to photons with energy larger than or equal to its band gap, nanostructured ZnO suggests that reactive oxygen species are formed as a result of the interaction of charge carriers (excitons) with dissolved oxygen and water molecules. However, due to the decrease in the generation of species brought on by the simpler recombination of excitons, the photocatalytic activity of un-doped ZnO-NPs remains incredibly low [18,19,20,21].

Several crucial attempts have been made to improve the photocatalytic performance of ZnO-NPs by lowering the exciton recombination and, as a result, the creation of super-reactive oxygen species. Several strategies have been developed to improve nanostructured ZnO’s photocatalytic performance by producing more of these species. Because it traps electrons and intensifies the separation of charge carriers, doping metallic ions with ZnO lattice is one of the intriguing methods for enhancing photocatalytic performance. Noble metals, such as silver (Ag), have been favored for the doping of ZnO because of their high reduction potential and improved photo-degradation capabilities. Because of its high reduction potential, doping of ZnO with Ag is best suited for preventing the recombination of charge carriers by trapping photo-excited electrons from the conduction band (CB), increasing the number of species that react with the pollutant and destroy it [14,22,23,24]. Furthermore, the nanocomposite faced another problem that came from the hindered aggregation effect that was produced by the high amounts of the nanostructured materials, leading to a decrease in the dangling bonds and reactivity, consequently reducing the photocatalytic performance. Therefore, the use of matrix material with a surface-to-volume ratio, such as carbon nanotubes (CNTs), could represent the optimum solution for solving this problem. [25,26,27,28,29,30].

Different techniques could be used to synthesize nanostructured materials to create nanocomposite structures. Pulsed laser ablation in liquid media (PLAL) is an effective, simple, and environmentally friendly process that can create NPs with a wide range of sizes and functionalities by only changing the laser’s parameters and the type of liquid medium. PLAL has been used to create a wide variety of nanoparticles, proving their adaptability. The benefits of this process include its simplicity, low cost, lack of vacuum champers, and ability to produce NPs without contamination in an incredibly clean and dependable manner. To produce a tiny particle with a distinct feature at the nanoscale, the PLA approach relies on the employment of high-intensity short pulses of up to a nanosecond from an infrared laser source in a concentrated environment [31,32,33,34,35,36,37,38].

In this article, we used the *eco*-friendly pulsed laser ablation in liquid media technique to synthesize ZnO nanoparticles and decorate CNTs to create the ZnO/MWCNTs nanocomposite, followed by using the same method to generate Ag nanoparticles interacting with ZnO/MWCNTs forming Ag-doped ZnO/MWCNTs. After that, XRD, SEM, EDX, XPS, and UV-Vis studies were used to characterize the produced NPs and nanocomposites. The ability of the created nanocomposite structure to eliminate organic pollutants, such as Rhodamine B, was next examined. For that, the novelty of the work was based on the use of a simple, *eco*-friendly, and green method to generate two different types of nanostructured materials (semiconducting ZnO and noble metal Ag) and embedding them to the matrix of CNTs to form the Ag-doped ZnO/MWCNTs nanocomposite. This combination between semiconductors and noble metals allows for enhancing the photocatalytic activity against organic pollutants, such as Rhodamine B.

## 2. Materials and Experimental Work

### 2.1. Preparation of Ag-Doped ZnO/MWCNTs Nanocomposite

**Figure 1:**
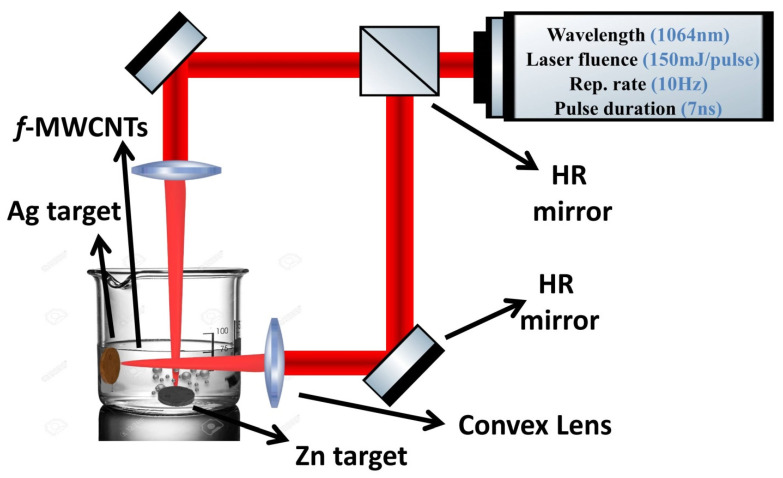
Schematic structure of preparation of Ag-doped ZnO/MWCNTs nanocomposite.

Zn pellet (Zn, BDH chemical Ltd. pool in UK) was used for the synthesis of ZnO nanostructured by pulsed laser ablation of cleaned Zn metal targets placed in a glass vessel filled with 5 mL of a solution of functionalized MWCNTs (f-MWCNTs) to produce the ZnO/MWCNTs nanocomposite, followed by repeating the previous procedure of pulsed laser ablation of another target, silver pellet (Ag, BDH chemical Ltd. pool in UK), immersed in the produced solution of the ZnO/MWCNTs nanocomposite. The functionalization procedure of MWCNTs was previously mentioned in the work of A. Mostafa et al. [39,40]. During this process, the laser beam of the Nd: YAG laser (λ = 1064 nm) with a 7 ns, a 10 Hz repetition rate, and 150 mJ of laser energy was focused on the surface of the target via convex lens for 10 min, as shown in the schematic diagram of the casting work in Figure 1.

### 2.2. Determination of Concentration of Nanostructured Materials

The amount of generation of the nanostructured material of ZnO or Ag can be simply estimated by using the following procedure. PLAL method was used to generate Ag NPs through ablation of an Ag plate. The amount of Ag NPs was related to the weight loss in the primary Ag’s precursor. So, the total number of Ag NPs was calculated as follows:

Weight of generated Ag = Weight of Ag_(before PLAL)_ − Weight of Ag_(after PLAL)_

By knowing the volume of the solution (10 mL), the concentration of generated Ag NPs can be simply calculated as 8.2 µg/L.

The generated nanocomposite was collected, cleaned with ultra-pure water, filtered, and dried at 50 °C to guarantee only calculated silver was linked to CNTs. The nano-composite was immersed in 15 mL of 70% nitric acid to quantify the total amount of coated Ag NPs on CNTs. So, Ag^+^ concentration could be calculated. Based on Ag NPs only on the CNTs’ outer surface, the loading amount of Ag in CNTs (W_Ag_) was determined by:

$${\mathrm{Weight}}_{\mathrm{decorated}\;\mathrm{Ag}\;\mathrm{on}\;\mathrm{CNTs}}=\frac{C_{\mathrm{Ag}+}V}{M-C_{\mathrm{Ag}+}V}$$

where C_Ag+_, V, and M are the concentration of Ag^+^ in the solution, the volume of the solution (250 mL), and the weight of the produced nanocomposite (5 mg) [41]. The concentration of Ag nanoparticles decorated on CNTs showed the decorated Ag NPs on the nanocomposite could be simply calculated as 7.7 µg/L.

### 2.3. Characterization Techniques

The crystalline structure of the prepared samples was characterized using an X-ray diffractometer (Shimadzu XRD 7000, Kyoto, Japan). The absorbance spectra of the prepared materials were studied by a UV–VIS-NIR spectrophotometer (JASCO 570 UV–VIS-NIR, Tokyo, Japan). The identification of the oxidation state was carried out by an X-ray photoelectron spectrometer (Thermo Scientific K-ALPHA instrument, East Grinstead, UK). The morphological analysis was carried out by a scanning electron microscope with a beam voltage of 20 kV associated with an EDX analyzer detector (PHILIPS/FEI QUANTA 250, Prague, Czech Republic) to study the chemical composition using TEAM^®^ software.

### 2.4. Adsorption Study

The degradation product, Rhodamine B (RhB, BDH Chemicals Ltd. pool in England), was used as a model pollutant for the degradation process, and the product’s photocatalytic activity was examined. As a UV light source in this investigation, a 40 W Xe lamp was used. In a typical experiment, the RhB solution was mixed with the photocatalyst. The suspension was discovered under light illumination after a 60 min dark adsorption period. The remaining RhB concentration was then measured using UV-visible absorption spectroscopy at 554 nm. Additionally, utilizing an appropriate amount of the photocatalyst (10–50 mg/L) with pH (pH = 3–11), contact time up to 140 min, and the starting concentration of RhB (5–110 mg/L), the photocatalytic performance of Ag-doped ZnO/MWCNTs was determined. Each experiment with a tested photocatalyst was repeated three times to evaluate the experimental errors. The following equation was used to compute the removal extent of RhB [42,43,44]:

Removal extent, percentage = (C_o_ − C_a_)/C_o_ × 100 (1)

where C_o_ and C_a_ are the concentration of the adsorbent material at the beginning of the adsorption process and after reaching the equilibrium of the adsorption process, respectively.

### 2.5. Desorption Study

To evaluate the reusability of native and modified sorbents in a successive adsorption and desorption process for a good number of recycles, we first carried out the RhB sorption under batch settings and then performed the desorption tests using 1 M of sodium hydroxide (NaOH) as the eluting agent. As with the sorption process, the desorption experiment begins with the addition of an appropriate amount of oven-dried sorbents to 100 mL of 1 M NaOH desorbing solution and then continues under conditions that are very similar to those of the sorption process. After that, the eluting agent was washed out of the regenerated sorbent with distilled water, and the sorbent was dried in a hot air oven. Once again, the same steps were used for a sorption–desorption cycle that may be repeated up to five times.

## 3. Results and Discussion

### 3.1. Investigation of the Prepared Ag NPs Doped ZnO/MWCNTs Nanocomposite

**Figure 2:**
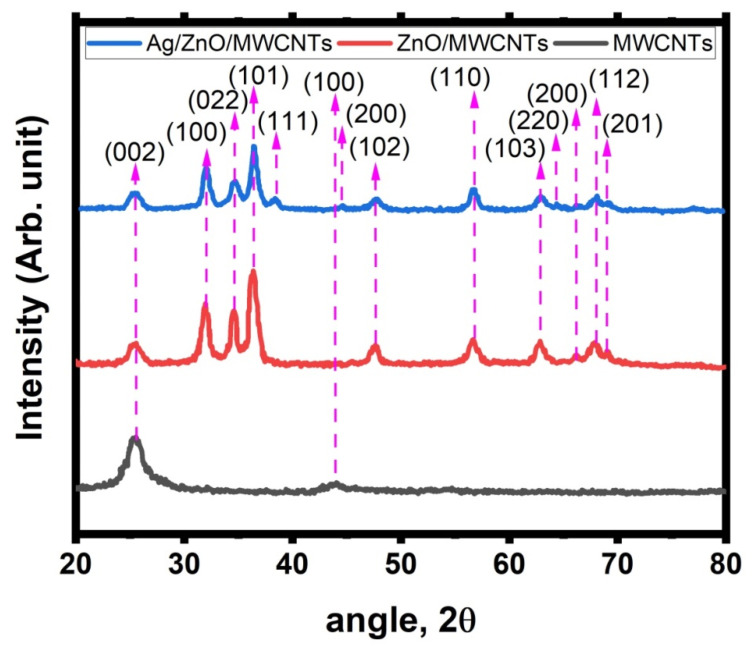
XRD diffractogram of MWCNTs, ZnO/MWCNTs, and Ag-doped ZnO/MWCNTs nanocomposite.

To verify the crystallinity and crystallite size, the XRD patterns of MWCNTs, ZnO/MWCNTs, and Ag NPs doped ZnO/MWCNTs nanocomposites were measured. The graphite structure of the MWCNTs is shown in Figure 2 along with how it developed in the diffraction patterns following embedding with ZnO NPs and then Ag NPs. The patterns demonstrate that the graphite structure coincided with the interplanar (002) of CNTs when the diffraction peaks at 26.10° occurred based on XRD card No. (PDF # 41-1487) [45]. Additionally, the diffractogram showed that MWCNTs decorated with ZnO NPs exhibit sharp peaks at 32.12°, 34.63°, 36.46°, 47.61°, 56.67°, 62.89°, 66.11°, 67.93°, and 69.01°. These diffraction peaks’ positions indicate the hexagonal wurtzite ZnO structure, since they correlate with the (1 0 0), (0 2 2), (1 0 1), (1 0 2), (1 1 0), (1 0 3), (2 0 0), (1 1 2), and (2 0 1) crystal planes, respectively, based on JCPDS card No. (36-1451) [46]. The diffractogram showed patterns with no additional diffraction peaks rather than a graphite structure and a ZnO structure. The sample was free of any impurities. The diffraction data clearly show that ZnO NPs had good crystallinity, with a predicted Scherer formula crystallite size of 22.5 nm. In other words, when Ag NPs were produced and doped with ZnO/MWCNTs, new diffraction peaks appeared at 38.41°, 44.47°, and 64.32°, respectively, corresponding to (111), (200), and (220), respectively, of the crystal faces of the silver structure, which was consistent with JCPDS card No. (65-2871) of the hexagonal wurtzite of the Ag structure [47]. It was concluded that the hybrids of Ag NPs doped ZnO/MWCNTs maintained the new crystal structure of the metal Ag, ZnO, and carbon nanotubes, which would be essential to guarantee the distinctive crystal structure and outstanding performance.

**Figure 3:**
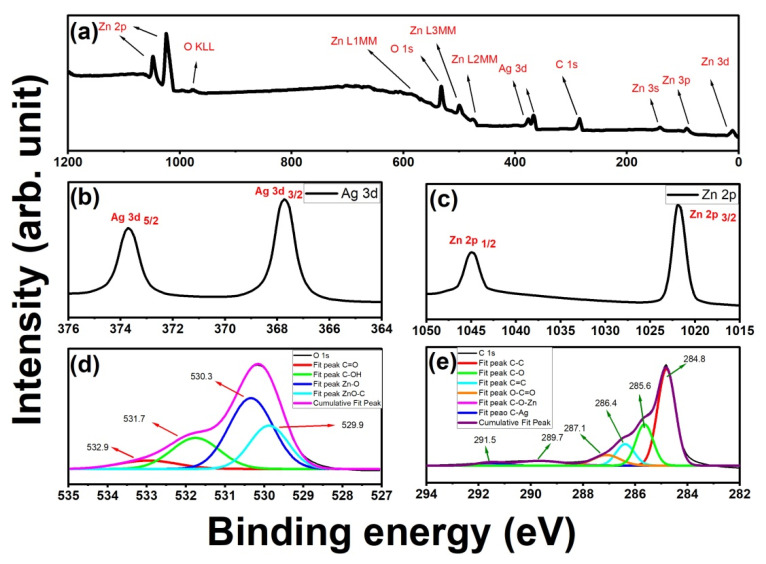
(**a**) A survey scan of XPS patterns of Ag-doped ZnO/MWCNTs nanocomposite and its HR spectra of (**b**) Ag 3d, (**c**) Zn 2p, (**d**) O 1s, (**e**) C 1s.

Using XPS, we were able to learn about the nature of the bonds in an Ag-doped ZnO/MWCNTs composite, as shown in the XPS survey spectra of the as-prepared sample in Figure 3a, where the Zn, Ag, O, and C core level peaks can be detected [48]. Figure 3b–e display the high-resolution XPS spectra of Ag 3d, Zn 2p, C 1s, and O 1s, respectively. In Figure 3b, the Ag 3d peak was deconvolved into two peaks by Gaussian fitting of Ag 3d_5/2_ and Ag 3d_3/2_ with binding energies of 368.2 and 373.3 eV, respectively, suggesting the existence of silver in the composite [48]. Additionally, as can be seen in Figure 3c, the Zn 2p spectrum was deconvolved into two peaks by Gaussian fitting (Zn 2p_3/2_ and Zn 2p_1/2_), which emerged at 1021.8 and 1048.8 eV, respectively, suggesting the existence of zinc in the composite [48]. Moreover, the O 1s peak was deconvolved into three peaks by Gaussian fitting of C=O, C-OH, Zn-O, and ZnO-C, as shown in Figure 3d, which are located at 529.9, 530.2, 531.7, and 532.9 eV, respectively. The presence of the CNTs’ backbone structure, as indicated by the first two tangled peaks, and the interaction of ZnO or Ag with the structure of the CNTs, as indicated by the third and fourth peaks, are both confirmed by the spectra. These XPS measurements, together with the microscopic findings, point to ZnO development occurring on the CNTS walls (presumably via the creation of Zn–O–C bonds) [49], as has been reported for metal/CNTs or metal oxide/CNT composite materials. Additionally, oxygen is likely directly bonded to the CNTS structure through the formation of strong covalent bonds between carbon and oxygen atoms (no Zn–C bonding was detected), as evidenced by the presence of oxygen components in the high-resolution XPS C 1s spectrum and the presence of carbon components in the O 1s spectrum [50,51]. According to Figure 3e, the high resolution of the C 1s peak was deconvolved into six peaks by Gaussian fitting located at 284.8, 285.6, 286.4, 287.1, 289.7, and 291.5 eV, which are attributed to the C-C, C-OH, C=C, O=C–O, C-O-Zn, and C-Ag groups. The first four peaks were related to the functionalized graphite structure of CNTs, while the last two peaks showed that ZnO and Ag interacted with the CNT skeleton [27,52,53]. Using their respective high-resolution XPS spectra, the amounts of the three precursor elements (C, O, Zn, and Ag) in the final composite structure were calculated. The atomic percentages of carbon, oxygen, zinc, and silver were found to be 40.67%, 32.22%, 18.17%, and 8.94%, respectively.

**Figure 4:**
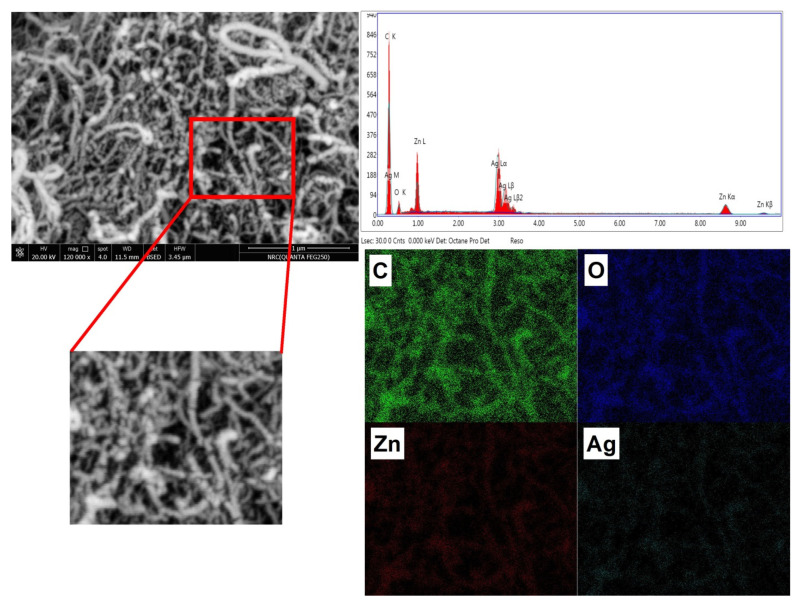
SEM image. EDX analysis and mapping of Ag-doped ZnO/MWCNTs nanocomposite.

Figure 4 is the image of the SEM morphological analysis and elemental analysis of MWCNTs, ZnO/MWCNTs nanocomposite, and Ag-doped ZnO/MWCNTs. It shows that the image of the MWCNTs sample has smooth tubular shapes of the carbon nanotube structure, while the image of the ZnO/MWCNTs nanocomposite shows the tubular shape of MWCNTs decorated with highly distributed particles grown on their outer shape, whereas the image of the Ag-doped ZnO/MWCNTs nanocomposite shows that the amount of the distribution of particles on the tubular structure was increased, and the rate of roughness was increased, which was certainly related to the ablation of Ag metals and the embedding of another nanostructured material from the Ag nanostructured material. Moreover, the energy-dispersive X-ray (EDX) spectroscopy was used to examine the chemical purity and elemental composition of MWCNTs, ZnO/MWCNTs, and Ag doped with ZnO/MWCNTs. The EDX spectrum of the produced Ag NPs doped with ZnO/MWCNTs. The binding energies of Zn are associated, while the binding energies of Ag and oxygen are related to ZnO. Moreover, the presence of C is related to CNTs. Therefore, the EDX spectrum confirms that the synthesized hybrid structure contains the components Ag, Zn, C, and O [54,55].

**Figure 5:**
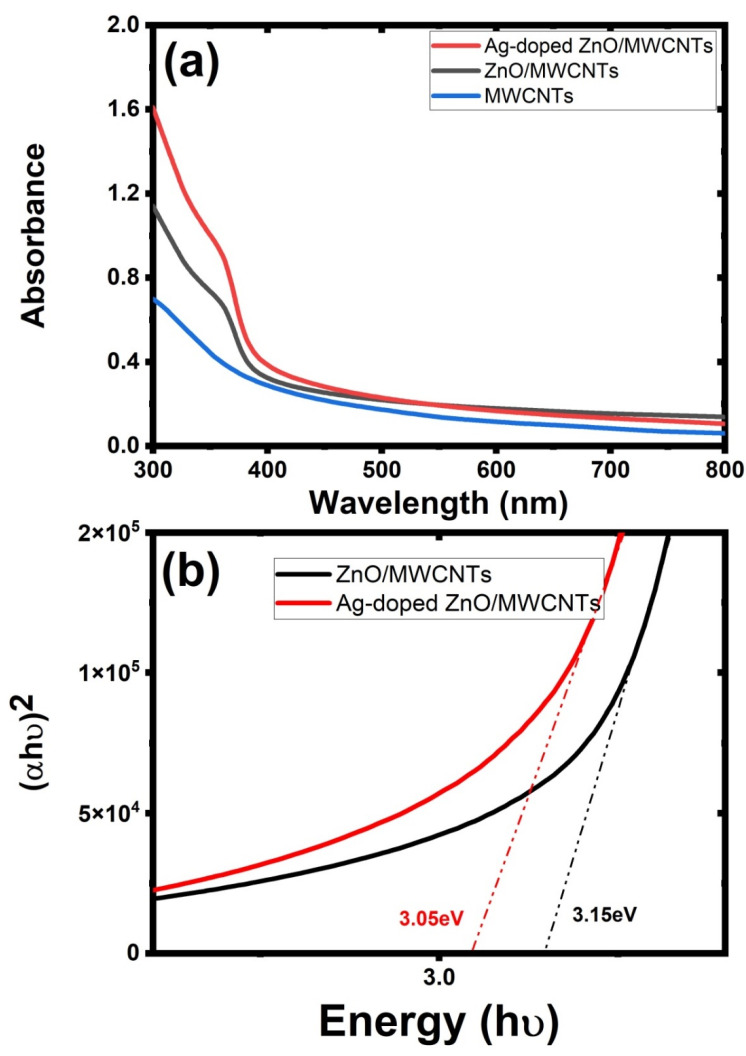
(**a**) Absorption spectrum of MWCNTs, ZnO/MWCNTs, and Ag-doped ZnO/MWCNTs nanocomposite and (**b**) their energy transition via Tauc relation.

The generated samples’ optical characteristics have a significant impact on the photocatalytic activity. In this investigation, the effect of Ag NP doping and ZnO NP surface decoration on the optical characteristics of ZnO/MWCNTs was assessed using UV-VIS-NIR spectroscopy. The absorption spectra of three samples are shown in Figure 5a, showing Ag NPs doped with ZnO/MWCNTs exhibit the maximum light absorption in the 400–700 nm regions. The following equation can be used to calculate the products’ band gaps [56]:

αhu = A(αhu − Eg)^n^

where α, hv, A, and E_g_ are the absorption coefficient, photon energy, constant, band gap energy, respectively. For a hybrid structure to function as an indirect transition semiconductor, n must equal 1/2. To calculate the energy gap, the plot of (hv)^1/2^ versus photon energy will be drawn. Pure ZnO/MWCNTs and ZnO/MWCNTs doped with Ag NPs had an estimated band gap of around 3.15 eV and 3.05 eV, respectively, as mentioned in Figure 5b. These findings support the doping of Ag NPs into the ZnO/MWCNTs matrix, since they demonstrate that adding Ag NPs causes the band gap of ZnO/MWCNTs to decrease. Based on the previous studies, this is because of the impurity that was introduced into the ZnO grains and may have trapped electrons stimulated from the conduction band, leading to a narrowing of the band gap and a continuous energy level [57].

### 3.2. Adsorption Process

#### 3.2.1. Affected Parameters of the Adsorption Process

The following test circumstances and parameters were used for optimizing the photocatalytic process, as 10–50 mg of the catalyst, 3–11 pH of the solution, 0–140 min of the reaction contact time, 5–110 mg/L of the RhB concentration, and 100 mL of the RhB comprised the reaction conditions.

**Figure 6:**
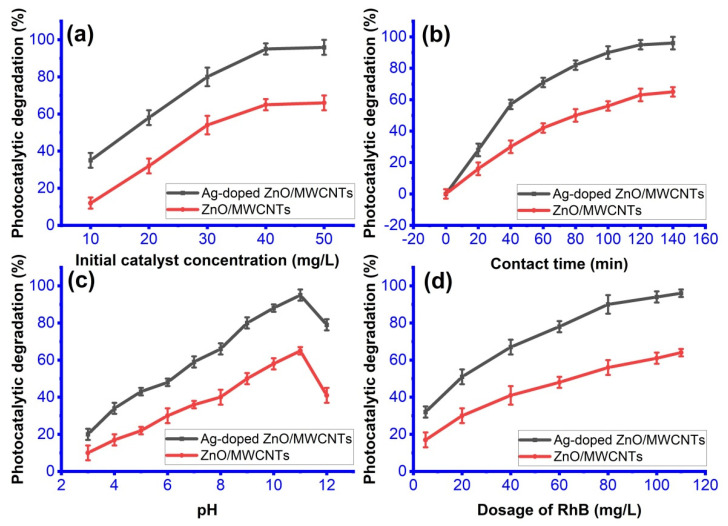
Effect of (**a**) initial catalyst concentration, (**b**) contact time, (**c**) pH, and (**d**) dosage amount of RhB on the removal of RhB.

The outcomes are displayed in Figure 6a. RhB was rapidly removed when the catalyst concentration was increased from 10 mg to 50 mg, rising from 12.44% and 35.29% of 10 mg to 40.01% and 95.53% of 40 mg for ZnO/MWCNTs and Ag-doped ZnO/MWCNTs, respectively. As the elimination percentage rises, the amount of pollutants that can be degraded per unit mass of the catalyst progressively reaches its maximum, and as the catalyst concentrations rise further, the ability to improve degradation efficiency is severely constrained. Therefore, in this experiment, a catalyst dosage setting of 40 mg is ideal [11,12].

In Figure 6b, by measuring the absorbance at 553 nm, the photocatalytic degradation of RhB utilizing the produced nanocomposite was assessed at various time intervals for 40 mg of the catalyst and at pH 10. With increasing irradiation time, a considerable decline in the RhB absorbance intensity at maximum 553 nm was seen, indicating a drop in dye concentration. According to a plot between the time and percent degradation efficiency of RhB, the deterioration effectiveness of RhB grows from 65.97% to 96.01% when the illumination time reaches 120 min and when the ZnO/MWCNTs decorated with Ag NPs is used [58,59,60,61].

According to research, industrial wastewater contains a wide range of pH values and plays a crucial role in the organic matter’s ability to decompose. Therefore, it is crucial to assess the effectiveness of the catalyst at various pH levels. A catalyst’s surface charge and the surface charge of organic dyes are both affected by pH, which also impacts how quickly the dye binds to the catalyst, and therefore, the rate of catalyst degradation. Therefore, using HCl and NaOH solutions to change the pH of the RhB solution, the impact of pH on the degradation process was investigated. Figure 6c demonstrates that the rate of degradation rose as the pH value increased, reaching a high (95%) at pH 11 (basic), starting to decline at pH 12 (80%), and reaching a minimum (20%) at pH 2 (acidic), which is also compatible with the literature. The catalyst and dye may repel each other electrostatically in acidic conditions where RhB occurs in cationic form (RhB^+^), which reduces the effect of dye degradation due to RhB^+^ being deprotonated when forming the zwitterion in basic media. So, the adsorption on the catalytic surface becomes difficult to be induced. In other words, at the fundamental conditions (above pH 11), the concentration of OH ions rises to the point where they cover the whole surface of the catalyst and leave it negatively charged. In this case, the adsorption process becomes easier due to the electrostatic attraction force produced from RhB^+^ and the negatively charged surface of the catalyst. So, the attraction of the RhB by the catalyst surface increases. Moreover, the radical ^•^OH is capable of being easily generated by hydroxide ions on the surface of the nanocomposite, leading to a logical enhancement of the photocatalytic process [62,63,64].

Different initial dye concentrations were used to study the impact of the initial dye concentration on the degradation efficiency of the synthesized nanocomposite (Figure 6d). The findings demonstrated that the degrading efficiency of the catalysts increased with increasing initial RhB concentration from 33.21% to 95.12% for Ag-doped ZnO/MWCNTs, with an increase in RhB from 5 mg/L to 110 mg/L, whereas it was noted that the rate was slightly increased with the concentration increase, and this may be related to two factors: (i) as the dye concentration is increased, a high number of RhB molecules will accumulate on the catalyst’s surface, preventing more of the dye molecules from reaching the surface; Second, (ii), when the dye molecules replicate, there will not be any available active sites for reaction. So, the increase rate would be slowed down [4,65,66,67].

#### 3.2.2. Kinetic Study

**Figure 7:**
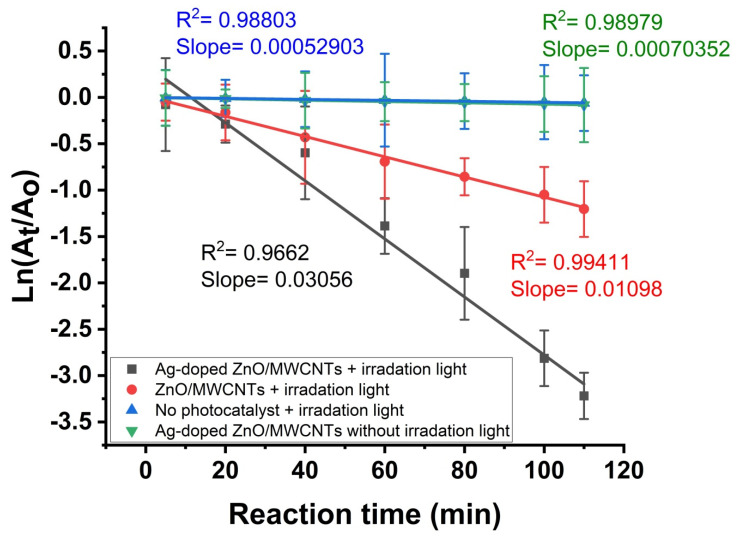
Absorption kinetic via pseudo-first order against RhB in the presence and absence of photocatalyst (ZnO/MWCNTs and Ag-doped ZnO/MWCNTs) and irradiation light.

The photocatalytic activity of ZnO/MWCNTs and their doping with Ag NPs to decompose RhB when exposed to light is depicted in Figure 7. After 120 min of ultraviolet exposure, compared to the pure ZnO/MWCNTs, Ag NPs doped MWCNTs have a higher photocatalytic degradation rate. MWCNTs, on the other hand, have a low photocatalytic activity in both regions. As a result, due to pure ZnO/MWCNTs having around 3.2 eV band gap, pure ZnO/MWCNTs can only be stimulated under UV light (376 nm). On the other hand, Ag NPs doped ZnO/MWCNTs exhibit improved photocatalytic capabilities in visible light, which is attributed to the narrowing of the band gap in comparison to pure ZnO/MWCNTs. Additionally, the photocatalytic activities of pure ZnO/MWCNTs and those doped with Ag NPs were compared using the results of the kinetic investigation. The reaction rate constants (k) can be determined by fitting the experimental data with the following pseudo-first-order reaction model [68,69,70]:

Ln (C_t_/C_o_) = kt

where the initial RhB concentration, the RhB concentration at time t, and the first-order rate constant are represented by C_o_, C_t_, and k, respectively. Figure 7 shows that the estimated data may be successfully fitted with the pseudo-first-order model. The rate constants for pure ZnO/MWCNTs and their decoration are 0.01098 min^−1^ and 0.03056 min^−1^, respectively, indicating the more significant photocatalytic activity of Ag-doped MWCNTs in comparison to the pure MWCNTs, while the degradation of RhB dye is almost negligible under UV light irradiation in the absence of the photocatalyst, demonstrating the potential of ZnO/MWCNTs before and after embedding with Ag as a catalyst for the photocatalytic degradation of organic dyes. Moreover, the concentrations of RhB remain almost constant and almost negligible throughout the experiment in the presence of the photocatalyst (Ag-doped ZnO/MWCNTs) and in the absence of the catalyst [71]. The performance of the Ag-doped ZnO/MWCNTs nanocomposite in comparison with some representative materials reported in previous works was tabulated in Table 1.

#### 3.2.3. Mechanism

**Figure 8:**
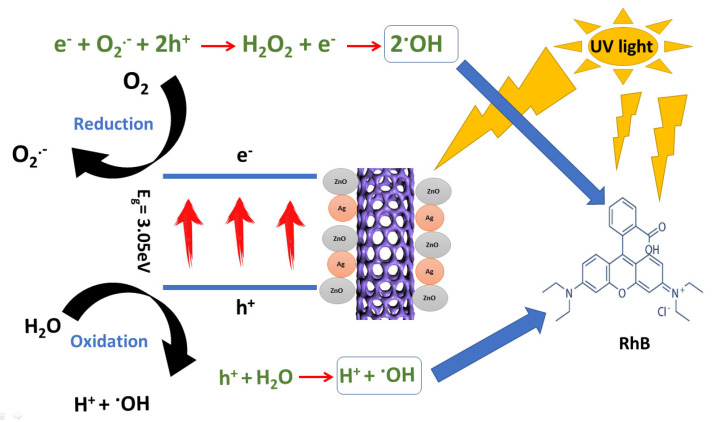
Schematic illustration of the photocatalytic mechanism of the photocatalyst for degradation of RhB.

Based on the experimental results, a suggested and schematic description of the photocatalytic activity of Ag NPs doped with ZnO/MWCNTs to destroy RhB under UV light was put forward. The pure ZnO/MWCNTs were unable to produce electron–hole pairs. The absorption of UV light generates electron–hole pairs in the nanocomposite particles. The holes react with the adsorbed H_2_O molecules to create ^•^OH, while the electron reduces O_2_ and generates O_2_^•^, H_2_O_2_, and ^•^OH. The superoxide radicals (^•^O_2_) and the photo-generated holes in the valence band (VB) have the potential to destroy RhB directly or indirectly by reacting with water to produce hydroxyl radicals (^•^OH). In addition, the photo-generated holes in the valence band (VB) have the potential to destroy RhB directly or indirectly by reacting with water to produce hydroxyl radicals (^•^OH) [73,74]. The radical-trapping experiment was mentioned in the schematic diagram of degradation of RhB in Figure 8.

#### 3.2.4. Reusability

**Figure 9:**
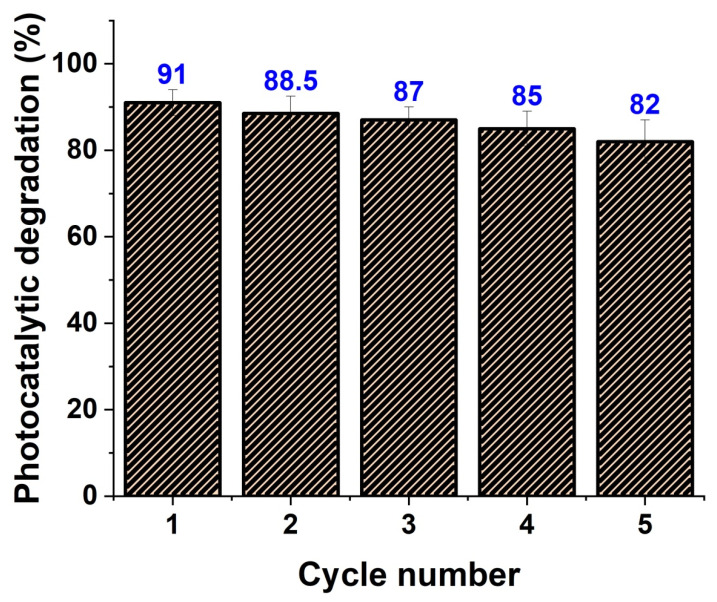
The usability of Ag-doped ZnO/MWCNTs as a catalytic degradable material against RhB.

In addition, the photocatalyst’s stability and usability are critical in real-world applications. As a result, after five consecutive cycles of UV irradiation (Figure 9), the photocatalytic performance of ZnO/MWCNTs with decorating Ag NPs was examined. The results showed that the photocatalytic performance of Ag NPs doped ZnO/MWCNTs was greater than 80% after five subsequent cycles, indicating its stability and reusability for practical applications. The losses in the degradation efficiency of the prepared nanocomposite in the reusability experiments were due to a decrease in the active sites on the nanocomposite’s surface from using NaOH, which peeled its active areas, leading to the tendency of the catalyst to agglomerate, mass loss, and some disintegration during the recovery process of the catalyst [75].

## 4. Conclusions

In conclusion, we successfully prepared a new and sophisticated form of ZnO/MWCNT embedded with Ag nanoparticles. A dual procedure of pulsed laser ablation in liquid medium was successfully used to fabricate Ag-doped ZnO/MWCNTs, which might be used as an RhB-degradable catalytic material. ZnO/MWCNTs were created using the PLA of a Zn target immersed in f-MWCNTs, which served as a solvent and matrix source for a further PLA of Ag immersed in the ZnO/MWCNTs solution that was created in the first PLA process. Notably, no extra reducing agents or surfactants were required in the synthesis. To characterize the produced materials, XRD, UV-vis, FE-SEM, XPS, and EDX were used. After that, the adsorption study showed that the effective RhB degradation from aqueous solutions was accomplished by the adsorbent, with an even better removal at low concentrations of about 40 mg/L of the adsorbent. According to the research, the adsorption is pH dependent, with pH 11 producing the greatest clearance. The removal process was controlled using pseudo-first-order rate kinetics. According to the adsorption results, RhB may be effectively removed from aqueous solutions using a hybrid composed of Ag-doped ZnO/MWCNTs.

## Figures and Tables

**Table 1:** The catalytic degradation of different organic pollutants over other nanocomposite-based Ag/ZO/MWCNTs catalysts.

Pollutant	Catalyst	K (min^−1^)	Physical Shape	Ref.
phenol	1%CNT loaded with ZnO-Ag	0.0045	powder	[72]
phenol	5%CNT loaded with ZnO-Ag	0.0057	powder	[72]
phenol	10%CNT loaded with ZnO-Ag	0.0068	powder	[72]
phenol	20%CNT loaded with ZnO-Ag	0.0051	powder	[72]
Methylene blue (MB)	CNTs loaded ZnO/Ag	0.0282	powder	[25]
RhB	Ag-doped ZnO/MWCNTs	0.03056	powder	This study

## Data Availability

Not applicable.
